# Case Report: Collagenous gastritis presenting with refractory dyspepsia and diffuse gastric atrophic-appearing changes

**DOI:** 10.3389/fmed.2026.1883727

**Published:** 2026-07-16

**Authors:** Yi-Ning Sun, Li Tang, Ying-Zhou Chen, Jin-Lin Yang, Zhu Wang

**Affiliations:** Department of Gastroenterology and Hepatology, West China Hospital, Sichuan University, Chengdu, Sichuan, China

**Keywords:** collagenous gastritis, gastric atrophy, hypogastrinemia, magnifying endoscopy, Masson trichrome staining, proton pump inhibitor, seronegative autoimmune gastritis

## Abstract

Collagenous gastritis (CG) is a rare fibroinflammatory disorder that may present with heterogeneous endoscopic findings. We report a 35-year-old woman with a 7-year history of refractory dyspepsia who had previously received a provisional diagnosis of seronegative AIG at another hospital because of *Helicobacter pylori*-negative, corpus-predominant gastric atrophy, iron deficiency, vitamin B12 deficiency, and concomitant thyroid autoimmunity. Persistent symptoms despite vitamin B12 and iron supplementation prompted referral to our center for further evaluation. Comprehensive reassessment revealed diffuse gastric atrophic-appearing changes, together with several findings discordant with established AIG, including persistent hypogastrinemia and magnifying endoscopic features inconsistent with typical AIG. Magnifying endoscopy with narrow-band imaging showed effacement of the surface microarchitecture and disorganized microvessels rather than the typical “cast-off skin” appearance of AIG, while acetic acid-enhanced imaging revealed residual central pits within the atrophic mucosa. Histopathological review with Masson trichrome staining demonstrated thickened subepithelial collagen bands with entrapped capillaries in both corpus and antral biopsies, confirming CG. After initiation of proton pump inhibitor therapy, the patient reported marked symptomatic improvement within 8 weeks. This case highlights CG as an overlooked differential diagnosis in unexplained diffuse gastric atrophic-appearing changes; nodular mucosal changes, atypical magnifying NBI findings, and subtle subepithelial pink material on routine histology should prompt collagen-specific staining to avoid diagnostic anchoring to more common forms of atrophic gastritis.

## Introduction

Collagenous gastritis (CG) is a rare fibroinflammatory disorder characterized by thickened subepithelial collagen deposition, mucosal inflammation, and epithelial injury (1–4). Although a subepithelial collagen band thicker than 10 μm is widely used as a histological hallmark, the diagnostic criteria for CG remain poorly standardized. Since its first description (1), CG has remained a poorly understood condition with heterogeneous clinical and endoscopic manifestations. Reported cases commonly emphasize gastric nodularity, abdominal pain, iron-deficiency anemia, diarrhea, or histological confirmation of collagen deposition (5–7). However, CG may also present with marked gastric atrophy, a phenotype that can overlap with more common causes of atrophic gastritis.

Autoimmune gastritis (AIG) is an immune-mediated disorder characterized by corpus-predominant oxyntic gland loss, iron and/or vitamin B12 deficiency, and, in established disease, reduced acid secretion with compensatory hypergastrinemia (8, 9). In clinical practice, *Helicobacter pylori*-negative corpus-predominant atrophy accompanied by micronutrient deficiency and autoimmune comorbidity may therefore lead to a provisional diagnosis of suspected seronegative AIG (9, 10). However, this diagnostic category should be reconsidered when gastric functional markers, autoantibody results, image-enhanced endoscopic findings, or histology are discordant with typical AIG (11).

Here, we report a case of CG in a patient who had previously been diagnosed with seronegative AIG because of gastric body atrophy described in an outside endoscopic report, micronutrient deficiency, and concomitant thyroid autoimmunity. Reassessment at our center revealed diffuse gastric atrophic-appearing changes that were not typical of established AIG and were not fully explained by conventional *H. pylori*-related atrophic gastritis. The case illustrates that persistent hypogastrinemia, negative AIG-related autoantibodies, atypical image-enhanced endoscopic findings, and collagen-specific staining can support diagnostic reclassification from presumed seronegative AIG to CG.

## Case report

A 35-year-old woman presented with chronic dyspepsia characterized by postprandial epigastric fullness, early satiety, and nausea for 7 years. One year before referral to our center, endoscopic evaluation at another hospital revealed marked gastric body atrophy. Because the patient had negative *H. pylori* testing, microcytic anemia, vitamin B12 deficiency, iron deficiency, and concomitant thyroid autoimmunity, she had been previously classified as having suspected seronegative AIG. However, the original high-resolution endoscopic images from the outside hospital were unavailable, and the images attached to the report were too limited for independent assessment of whether the endoscopic pattern fulfilled typical AIG features. Baseline laboratory testing showed microcytic anemia with hemoglobin of 106 g/L and mean corpuscular volume of 78 fL, severe vitamin B12 deficiency of 148 pg./mL, hypoferritinemia of 12.86 μg/L, and unexpectedly low serum gastrin of 10.7 pg./mL. Thyroid peroxidase antibody and thyroglobulin antibody levels were elevated at 121 IU/mL and 176 IU/mL, respectively. *H. pylori* testing by urea breath test and serum antibody testing was negative. Anti-parietal cell antibody and anti-intrinsic factor antibody were also negative. The patient received vitamin B12 and iron supplementation, prokinetic agents, and mucosal protective therapy. Proton pump inhibitors were not used at that time because the clinical focus was placed on suspected AIG-related atrophy and micronutrient deficiency rather than acid-mediated epithelial injury.

**Figure 1:**
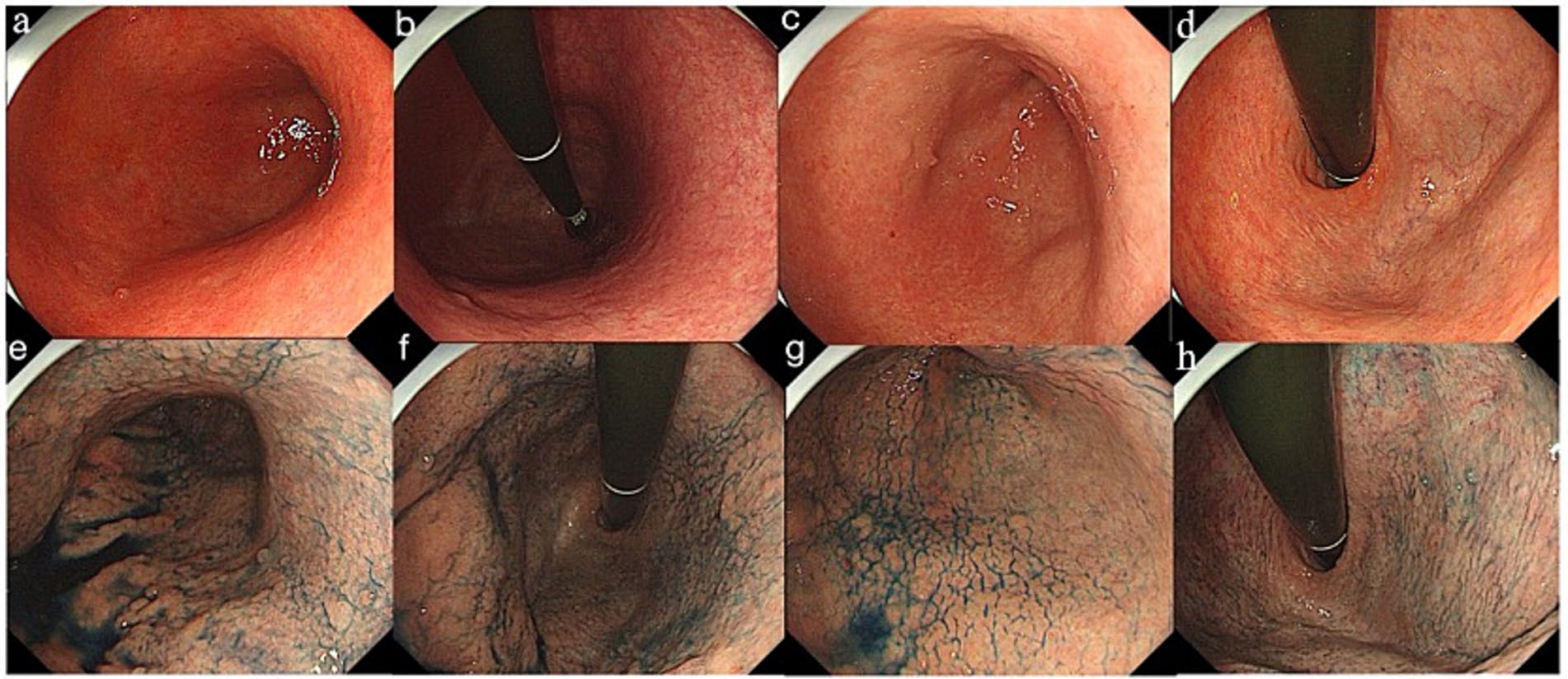
Conventional white-light and indigo carmine chromoendoscopic findings. **(a–d)** White-light images; **(e–h)** indigo carmine chromoendoscopic images. Repeat endoscopy at our center showed diffuse rough, thinned, atrophic-appearing mucosa, with loss of folds and irregular surface topography. Indigo carmine chromoendoscopy highlighted nodular or uneven mucosal changes along the greater curvature of the corpus, especially in panel e. The antral mucosa appeared slightly rough, with possible subtle nodular changes on white-light imaging **(c)**, although these were not clearly accentuated after chromoendoscopy **(g)**. **(a,b,e,f)** body **(c,g)** antrum **(d,h)** fundus.

Despite 12 months of this regimen, her dyspeptic symptoms persisted without appreciable improvement, and she was referred to our center for further evaluation. Follow-up endoscopy revealed diffuse rough, thinned, atrophic-appearing mucosa involving both the corpus and antrum, with loss of gastric folds, diffuse mucosal coarseness, a mosaic-like pattern, and irregular surface topography accentuated by indigo carmine chromoendoscopy (Figure 1). In particular, indigo carmine chromoendoscopy highlighted nodular or uneven mucosal changes along the greater curvature of the corpus (Figure 1e). The antral mucosa appeared slightly rough, and possible subtle nodular mucosal changes were suspected in the anterior wall of the antrum on white-light imaging (Figure 1c), although these changes were not clearly accentuated after chromoendoscopy (Figure 1g). Repeat laboratory evaluation showed persistently suppressed gastrin-17 of 0.40 pmol/L, markedly reduced pepsinogen I of 3.0 μg/L, suboptimal ferritin response after supplementation of 21.4 μg/L, and partial correction of vitamin B12 to 393 pg./mL. Taken together, the diffuse atrophic-appearing changes were not typical of established AIG and were difficult to attribute to conventional *H. pylori*-related atrophic gastritis given negative *H. pylori* testing; persistent hypogastrinemia, together with negative AIG-related autoantibodies, prompted further image-enhanced endoscopic and histopathological reassessment.

**Figure 2:**
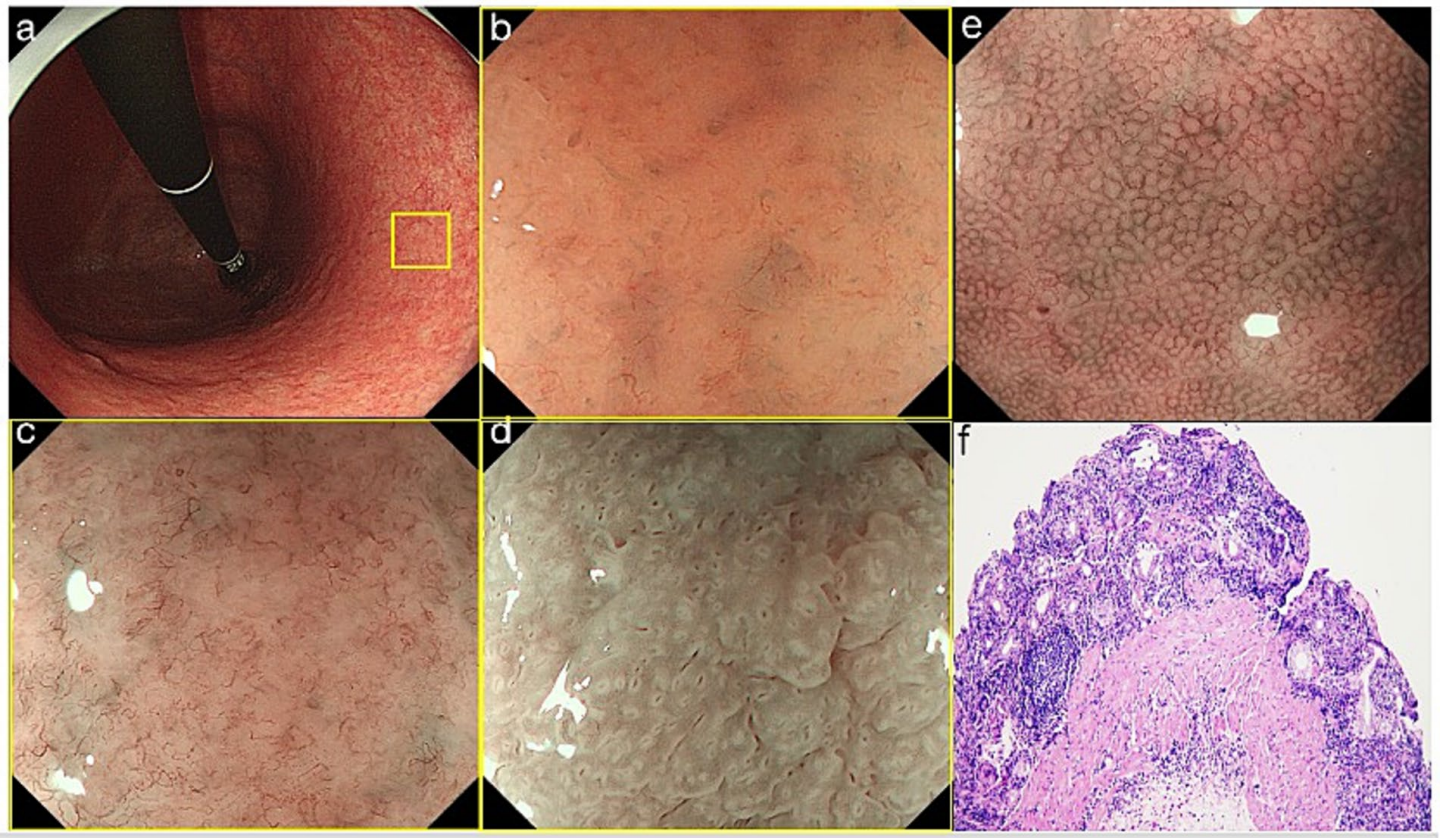
**(a)** Marked atrophy of the gastric mucosa is recognized in the lesser curvature of the corpus. **(b–d)** Magnified image of the lesser curvature (yellow box), surface microstructure is blurred, microvessels are disarranged, acetic acid staining reveals preserved central pits of fundic gland microstructures. **(b)** white light image **(c)** narrow-band image **(d)** acetic acid staining. **(e)** Representative magnifying endoscopic features of autoimmune gastritis showing a polygonal vascular network with loss of central pits (“cast-off skin” appearance). **(f)** Biopsy of gastric body shows inflammatory cell infiltration, destruction of intrinsic glands with pseudopyloric metaplasia, and subtle subepithelial pink material (HE×100).

**Figure 3:**
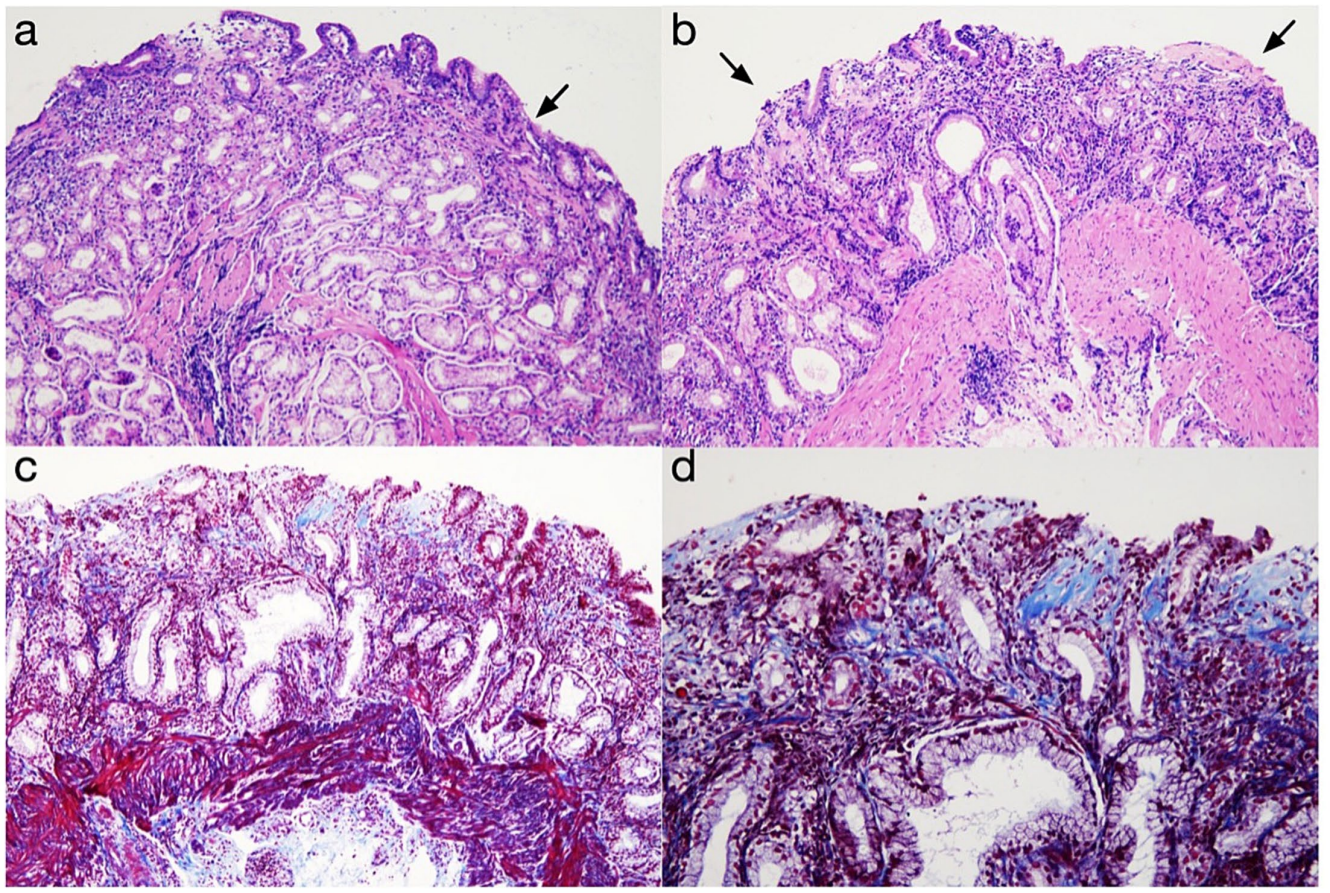
Histological evaluation **(a,b)** Biopsies of antrum and gastric body show subepithelial pink material (arrows, HE×100). **(c,d)** Masson staining confirms the pink material as subepithelial collagen deposition (×100, ×200).

Magnifying endoscopy with narrow-band imaging (NBI) provided further diagnostic information. Instead of a regular polygonal microvascular pattern with loss of central pits, often described as a “cast-off skin” appearance in AIG (Figure 2e), the lesion in this case showed effacement of the surface microarchitecture and disorganized microvessels (Figures 2b,c). Acetic acid-enhanced imaging demonstrated residual central pits within the atrophic mucosa (Figure 2d), further suggesting that the atrophic appearance (Figure 2a) was not fully explained by typical autoimmune oxyntic gland destruction. Histopathology from gastric biopsies initially showed chronic inflammatory infiltrates, pseudopyloric metaplasia, near-complete loss of specialized oxyntic glands, and focal intestinal metaplasia (Figure 2f). Because the clinical, serological, magnifying endoscopic findings, and subtle subepithelial pink material on routine hematoxylin and eosin staining were discordant with established AIG, the biopsy specimens were reassessed with Masson trichrome staining. This revealed thickened subepithelial collagen bands with entrapped capillaries in both corpus and antral biopsies, accompanied by lymphocytic infiltration at the lamina propria-collagen interface and focal surface epithelial detachment (Figure 3). These findings established the diagnosis of CG. The revised diagnosis of CG guided a therapeutic shift. Prokinetic agents and mucosal protectants were used as needed, and esomeprazole 40 mg daily was initiated. Within 8 weeks, the patient reported marked symptomatic improvement, with the visual analog scale score for dyspepsia decreasing from 8/10 to 3/10. Meal tolerance improved, body weight stabilized, and hemoglobin increased to 118 g/L. Endoscopic surveillance was scheduled within 6–12 months.

The diagnostic course can be summarized as follows: long-standing refractory dyspepsia; previous classification as suspected seronegative AIG at another hospital based on reported gastric body atrophy and micronutrient deficiency; persistent symptoms despite supplementation; reassessment at our center showing diffuse gastric atrophic-appearing changes, hypogastrinemia, chromoendoscopic nodular or uneven mucosal changes, and atypical magnifying endoscopic findings; collagen-specific staining confirming CG; and symptomatic improvement after proton pump inhibitor therapy.

## Discussion

This case highlights CG as an underrecognized differential diagnosis in patients with unexplained diffuse gastric atrophic-appearing changes. Its clinical value lies not only in the rarity of CG, but also in the diagnostic pathway it illustrates: persistent hypogastrinemia and discordant magnifying endoscopic findings prompted collagen-specific staining and led to diagnostic reclassification. The previous classification of suspected seronegative AIG at another hospital was clinically understandable because the outside endoscopic report described gastric body atrophy, and the patient also had iron deficiency, vitamin B12 deficiency, and thyroid autoimmunity. These features may raise clinical suspicion for autoimmune-mediated oxyntic gland loss. However, typical AIG could not be independently confirmed from the limited outside images, and repeat endoscopy at our center showed diffuse atrophic-appearing changes involving both the corpus and antrum, which were neither typical of established AIG nor readily explained by conventional *H. pylori*-related atrophic gastritis given negative *H. pylori* testing. Moreover, persistent hypogastrinemia was discordant with the physiological pattern usually expected in established AIG, in which corpus oxyntic gland destruction and hypochlorhydria typically lead to compensatory hypergastrinemia (8–11). Therefore, in patients with unexplained diffuse gastric atrophic-appearing changes provisionally attributed to seronegative AIG, low or persistently suppressed gastrin together with atypical image-enhanced endoscopic findings should be regarded as practical clues prompting histopathological reassessment and collagen-specific staining, rather than as evidence supporting AIG. To clarify the differential diagnostic framework illustrated by this case, the key clinical, serological, endoscopic, histopathological, and therapeutic distinctions between AIG and CG are summarized in Table 1.

**Table 1:** Comparison between autoimmune gastritis and collagenous gastritis.

Feature	Autoimmune gastritis (AIG)	Collagenous gastritis (CG)
Age/Sex	Middle-aged to elderly adults; female predominance	Typically affects younger individuals (median age: ~18 years); female predominance
Symptoms	Dyspepsia and anemia (iron deficiency anemia [IDA] and/or pernicious anemia [PA]); IDA usually precedes PA; approximately 30% present with both IDA and PA	Chronic abdominal pain and anemia (~12%); vitamin B12 deficiency is uncommon
Pathogenesis	Autoimmune destruction of parietal cells and gastric H^+^/K^+^-ATPase via CD4^+^ T-cell mediated cytotoxicity, inducing parietal cell apoptosis and mucosal atrophy	Subepithelial collagen deposition (>10 μm) with fibroblast activation; likely immune-mediated or drug/infection-related
Serological Features	Positive anti-parietal cell antibodies (60–85%) and anti-intrinsic factor antibodies (50–60%); hypergastrinemia; decreased pepsinogen I; decreased PGI/PGII ratio	Typically negative for autoimmune antibodies; gastrin levels normal or decreased
Endoscopic Features	Atrophic mucosa with visible submucosal vessels (predominantly in the corpus/fundus); loss of rugal folds; corpus intestinal metaplasia; possible polyps or type 1 neuroendocrine tumors (NETs)	Gastric mucosal nodularity (in >50% of cases); occasional mucosal atrophy; corpus-predominant or diffuse distribution; intestinal metaplasia and NETs are rare
Magnifying Endoscopy Findings	Preserved polygonal microvascular pattern with pit loss; surface architecture relatively uniform despite glandular atrophy	Disorganized vascular pattern with irregular or partially preserved pits; acetic acid highlights residual central pits
Histologic Features	Glandular atrophy, intestinal metaplasia, and enterochromaffin-like (ECL) cell hyperplasia	Thickened subepithelial collagen band (>10 μm) with entrapped capillaries (‘trapped capillaries’) and epithelial detachment; inflammatory infiltrate; ECL cell hyperplasia typically absent
Inflammatory Pattern	Mononuclear inflammatory infiltrate	Three histologic patterns: atrophic (most common), lymphocytic gastritis-like, or eosinophil-rich
Special Stains	Chromogranin A positive (ECL hyperplasia); collagen stains negative	Masson trichrome and tenascin positive; type IV collagen negative
Associated Conditions	Other autoimmune disorders (e.g., autoimmune thyroid disease, type 1 diabetes)	Collagenous colitis/sprue, celiac disease, autoimmune disorders, immune deficiencies, or olmesartan exposure
*Helicobacter pylori*	May act as a trigger via molecular mimicry between bacterial and parietal cell antigens	*H. pylori* infection reported in ~10% of CG cases
Treatment	Vitamin B12 and Iron supplementation; management of complications	PPIs, Corticosteroids, immunomodulators, and removal of potential triggers (e.g., gluten-free diet, drug withdrawal)
Prognosis	Chronic course with risk of developing neuroendocrine tumors	Variable; chronic but generally benign course, with rare histologic remission

Conventional and chromoendoscopic findings provided an important first clue. Indigo carmine chromoendoscopy highlighted nodular or uneven mucosal changes along the greater curvature of the corpus, with possible subtle nodularity in the antrum. These findings required careful interpretation. Although gastric mucosal nodularity is a well-described endoscopic finding in CG (2, 5, 7, 12, 13), nodular or polypoid lesions are not specific to CG and may also be encountered in AIG, including hyperplastic polyps, pseudopolypoid lesions, or relatively preserved oxyntic mucosa within an atrophic background (14, 15). Therefore, in the present case, the nodular or uneven mucosal changes highlighted by chromoendoscopy were not interpreted in isolation. Rather, they were considered together with persistent hypogastrinemia, negative AIG-related autoantibodies, atypical magnifying endoscopic findings, and subsequent histological evidence of collagen deposition.

Magnifying endoscopy provided a second layer of diagnostic information. Image-enhanced endoscopy in AIG may show a polygonal microvascular pattern with pit loss, sometimes described as a “cast-off skin” appearance (11, 15). In contrast, our case showed effacement of the surface microarchitecture and disorganized microvessels on NBI, while acetic acid-enhanced imaging demonstrated residual central pits. These findings suggested that the atrophic appearance was not simply attributable to autoimmune oxyntic gland destruction, but may reflect epithelial injury and subepithelial remodeling. This interpretation was supported by Masson trichrome staining, which revealed thickened subepithelial collagen bands with entrapped capillaries in both corpus and antral biopsies. In retrospect, the hematoxylin and eosin-stained section shown in Figure 2f already demonstrated subtle subepithelial pink material, consistent with the more clearly illustrated subepithelial collagen deposition in Figures 3a,b. However, because Figure 2f was originally selected to emphasize inflammatory cell infiltration, destruction of intrinsic glands, and pseudopyloric metaplasia, the subepithelial collagen-like material was not highlighted. This observation underscores that collagen deposition may be present but underrecognized on routine hematoxylin and eosin staining when the diagnostic focus is placed on atrophy and metaplastic changes rather than CG.

Histopathological recognition of CG requires vigilance. Routine hematoxylin and eosin staining may show only chronic inflammation, epithelial injury, or subepithelial eosinophilic material, and the collagen band can be overlooked if CG is not specifically considered (2, 5, 12). Collagen-specific stains, including Masson trichrome and Sirius red staining, are therefore useful when unexplained gastric atrophy, refractory symptoms, or discordant serological and endoscopic findings are present (2, 12). In this case, collagen-specific staining transformed the diagnostic interpretation from suspected seronegative AIG to CG.

Nevertheless, CG remains diagnostically challenging because standardized diagnostic criteria have not been fully established. Although a subepithelial collagen band thicker than 10 μm is widely used as a practical histological hallmark, collagen deposition may be patchy or diffuse, and the accompanying inflammatory pattern may vary. Therefore, the diagnosis should integrate collagen thickness, collagen distribution, epithelial injury, inflammatory features, endoscopic appearance, and clinical context. This uncertainty is directly relevant to the present case, in which atrophic changes and micronutrient deficiency initially favored suspected seronegative AIG, whereas collagen-specific staining revealed the defining abnormality of CG (2, 5, 12, 13).

The pathogenesis of CG remains incompletely understood, but available evidence supports an immune-mediated and fibroinflammatory model rather than simple passive collagen accumulation. Reported associations with autoimmune diseases, celiac disease, immune deficiency, collagenous colitis or sprue, and medication-related mucosal injury suggest that persistent mucosal immune activation may contribute to epithelial injury and abnormal repair. More recent studies have further implicated activated or exhausted lymphocytes, altered CD4/CD8 T-cell balance, reduced tissue-resident T cells, enhanced collagen-gene expression by fibroblasts, and mucosal-homing signals in CG pathogenesis (16, 17). Together, these findings support a plausible sequence in which immune-mediated epithelial injury promotes fibroblast activation and excessive subepithelial collagen deposition. However, these mechanisms remain incompletely validated, and future immune or stromal biomarkers may help establish more objective diagnostic criteria. In our patient, concomitant thyroid autoimmunity and lymphocytic inflammation were compatible with an immune-mediated background, whereas the absence of AIG-related autoantibodies and persistent hypogastrinemia argued against established AIG as the primary diagnosis.

The distinction between CG and AIG has practical therapeutic implications. In established AIG with marked oxyntic gland loss, long-term acid suppression may have limited physiological rationale and should be considered carefully, particularly in patients with hypergastrinemia or enterochromaffin-like cell hyperplasia. By contrast, acid suppression has been used empirically in CG to reduce acid-mediated epithelial injury and may help interrupt the injury-repair cycle contributing to collagen deposition. However, standardized treatment for CG remains lacking, and PPIs should not be regarded as an established first-line therapy because responses are variable and many patients do not achieve substantial or durable benefit with acid suppression alone (2, 7, 13, 18). Other reported therapeutic options include topical budesonide, systemic corticosteroids, immunomodulators, nutritional support, and removal of potential triggers when identifiable (13, 18). In particular, topical budesonide has shown clinical and histological benefit in a retrospective open-label cohort, further supporting the biological heterogeneity of CG and the need for individualized treatment (18). In our patient, esomeprazole therapy was followed by marked symptomatic improvement within 8 weeks. This favorable response supports the clinical relevance of diagnostic reclassification but should not be generalized to all patients with CG. It may reflect a specific phenotype in which acid-mediated epithelial injury contributed to symptoms or mucosal damage, possibly related to the unusual diffuse atrophic-appearing phenotype. Continued clinical and endoscopic follow-up is needed to determine whether symptomatic improvement is accompanied by histological improvement.

The cause of vitamin B12 deficiency in this patient remains uncertain. Although vitamin B12 deficiency initially supported the suspicion of AIG, it was unlikely to be attributable to established AIG because AIG-related autoantibodies were negative and serum gastrin was persistently suppressed. Vitamin B12 deficiency has rarely been described in patients with CG, including a reported adult case of collagenous gastritis with severe atrophy and negative AIG-related antibodies (19). However, the limited number of reports does not establish a direct causal relationship between CG and vitamin B12 deficiency. In the present case, long-standing dyspepsia with reduced meal tolerance, chronic gastric inflammation, severe oxyntic gland loss, and possible non-AIG-related nutritional or malabsorptive factors may have contributed (20).

This case provides a clinically actionable message for physicians managing patients provisionally classified as having suspected seronegative AIG. Gastric atrophic changes accompanied by iron deficiency, vitamin B12 deficiency, and autoimmune comorbidity should not be automatically labeled as AIG when gastric functional markers and image-enhanced endoscopic findings are discordant. In such cases, CG should be considered, particularly when chromoendoscopy shows nodular or uneven mucosal changes that are not fully consistent with typical AIG and when routine histology suggests subtle subepithelial eosinophilic or pink material. Persistent hypogastrinemia, discordant magnifying endoscopic findings, chromoendoscopic nodularity, and Masson or Sirius red staining may serve as a practical set of diagnostic clues, rather than a definitive diagnostic triad, for reclassifying presumed seronegative AIG in patients with unexplained diffuse gastric atrophic-appearing changes and guiding individualized management.

## Data Availability

The original contributions presented in the study are included in the article/supplementary material, further inquiries can be directed to the corresponding author.
